# Combined Analysis of Grade Differences in Lapsang Souchong Black Tea Using Sensory Evaluation, Electronic Nose, and HS-SPME-GC-MS, Based on Chinese National Standards

**DOI:** 10.3390/foods13213433

**Published:** 2024-10-28

**Authors:** Xiaomin Pang, Zi Yan, Jishuang Zou, Pengyao Miao, Weiting Cheng, Zewei Zhou, Jianghua Ye, Haibin Wang, Xiaoli Jia, Yuanping Li, Qi Zhang

**Affiliations:** 1 Center for Information Technology and Laboratory Management, Wuyi University, Wuyishan 354300, China; pxm0601@wuyiu.edu.cn; 2 College of Tea and Food Science, Wuyi University, Wuyishan 354300, Chinaa13883402655@163.com (J.Z.); 5220831031@fafu.edu.cn (P.M.); 5220330096@fafu.edu.cn (W.C.); 5220831088@fafu.edu.cn (Z.Z.); jhye1998@wuyiu.edu.cn (J.Y.); jiaxl2010@wuyiu.edu.cn (X.J.); li343000@126.com (Y.L.); 3 College of Resources and Environment, Fujian Agriculture and Forestry University, Fuzhou 350002, China; 4 College of Horticulture, Fujian Agriculture and Forestry University, Fuzhou 350002, China; 5 College of Life Sciences, Longyan University, Longyan 364012, China

**Keywords:** Lapsang Souchong black tea, grade, Chinese national standards, electronic nose, HS-SPME-GC-MS

## Abstract

Tea standard samples are the benchmark for tea product quality control. Understanding the inherent differences in Chinese national standards for Lapsang Souchong black tea of different grades is crucial for the scientific development of tea standardization work. In this study, Lapsang Souchong black tea of different grades that meet Chinese national standards was selected as the research object. The aroma characteristics were comprehensively analyzed through sensory evaluation, electronic nose, and HS-SPME-GC-MS (headspace solid-phase microextraction gas chromatography–mass spectrometry). The findings indicate that the higher-grade Lapsang Souchong has a higher evaluation score. The results of electronic nose analysis indicate that the volatiles with differences in tea of different grades were mainly terpenoids and nitrogen oxides. The results of HS-SPME-GC-MS analysis show that the odor characteristics of the super-grade samples are mainly floral and fruity, and these substances mainly include D-Limonene, 3,7-dimethyl-1,6-octadien-3-ol and 3-Hydroxymandelic acid, and ethyl ester. The primary aroma characteristics of the first-grade samples are floral, sweet, woody, and green, with key contributing compounds including 2-Furanmethanol, 1-Octen-3-ol, and 5-ethenyltetrahydro-α,α,5-trimethyl-cis-, 4,5-di-epi-aristolochene. The main aroma characteristics of the second-grade samples are green, herbal scent, and fruity, and the main substances include 3,7-dimethyl-1,6-octadien-3-ol, 2,3-dimethylthiophene, Dihydroactinidiolide, and Naphthalene-1-methyl-7-(1-methylethyl)-. It is worth noting that the second-grade samples contain a large amount of phenolic substances, which are related to the smoking process during processing. This study lays a solid foundation for the preparation of tea standard samples and the construction of the tea standard system.

## 1. Introduction

Black tea is one of the most popular beverages around the world, celebrated for its unique flavor, rich aroma, and numerous health benefits [1]. Its widespread appeal has made it a cornerstone of social customs and daily life in various cultures over centuries. Among the diverse varieties of black tea, Lapsang Souchong is particularly notable for its distinctive taste. This tea is recognized for its strong, sweet flavor complemented by a fruity aroma, and it possesses a rare smoky note that sets it apart from other types of tea. This characteristic flavor is a result of traditional processing methods, where the leaves are smoked over pinewood fires, creating a profile that has enchanted tea lovers globally [2]. Originating in the Wuyi Mountains of Fujian Province, China, Lapsang Souchong is historically significant as it is considered the world’s first black tea.

As the global interest in high-quality tea varieties increases, distinguishing between the different grades of Lapsang Souchong has become essential. Establishing national tea standards necessitates an understanding of the material basis for these grades, providing a theoretical foundation for classification. Such distinctions are crucial for ensuring product quality and enhancing competitiveness in the global market, where consumers are increasingly willing to pay premium prices for exceptional teas.

Aroma is a key quality attribute in black tea, influencing its grading and overall sensory appeal. The aroma profile serves as both an indicator of quality and a reflection of the tea’s processing methods and raw materials [3]. Volatile compounds are the primary contributors to the aroma and are essential for evaluating the product’s quality. Advanced techniques, such as electronic nose systems and gas chromatography–mass spectrometry (GC-MS), have been effectively utilized to identify these aromatic components in various foods, including tea, wine, and rice [4,5,6]. The volatile compounds typically found in Lapsang Souchong include alcohols, phenols, aldehydes, olefins, ketones, esters, and ethers, each adding to its complex flavor [2].

Numerous studies have examined the aroma characteristics of Lapsang Souchong black tea. Li et al. [7] studied the mechanism of the formation of aroma in Lapsang Souchong black tea by the smoking process and found that the smoking process would promote phenolic odor substances in Lapsang Souchong black tea. Xiao et al. [8] used GC-MS and GC-O to jointly analyze the aroma differences between Lapsang Souchong black tea and other black teas. At present, there is still a lack of systematic research on the aroma characteristics of standard samples of Lapsang Souchong black tea of different grades. This absence hinders effective quality control measures and limits the scientific advancement of Lapsang Souchong black tea’s quality evaluation.

Standardized tea samples are crucial in the tea industry, acting as benchmarks for quality and consistency. They provide reference points for flavor characteristics and support the development of new products. In this context, analyzing national standard samples of Lapsang Souchong—ranging from super-grade to second-grade—is vital. The objective of this research is to identify the volatile compounds that play a role in grading Lapsang Souchong within the framework of China’s national standard system. Utilizing sensory evaluation, electronic nose analysis, and HS-SPME-GC-MS, this study aims to identify the key volatile compounds that differentiate the grades of this tea.

Moreover, advanced analytical techniques, such as heat map analysis, principal component analysis (PCA), and orthogonal partial least squares discriminant analysis (OPLS-DA), will offer a solid framework for identifying characteristic volatile compounds. These methods are crucial for establishing a theoretical basis for both the Chinese tea standard system and the international tea industry, where such research can contribute to global quality standards.

## 2. Materials and Methods

### 2.1. Sample Collection

Three different grades of national standard Lapsang Souchong black tea samples—super-grade (A), first-grade (B), and second-grade (C)—were sourced from the Wuyishan Market Supervision Bureau and the Wuyishan Tea Industry Association. These samples conform to the standard number DB35/T1838-2022 and are valid from 23 February 2022 to 22 February 2025. The selection of samples was conducted rigorously according to the sensory quality criteria specified in the national standard “Tea-Sensory Evaluation Method” (GB/T 23776-2018 [9]). The tea leaves were ground using a 0.2 mm sieve, with some portions stored at −20 °C for subsequent experiments.

### 2.2. Experimental Reagents

Ethyl decanoate (≥99%) purchased from Sigma-Aldrich (Shanghai, China) was used as the internal standard substance.

### 2.3. Sensory Evaluation

Sensory evaluation were carried out in line with the national standard method GB/T 23776-2018 “Tea-Sensory Evaluation Method.” Ten experienced tea tasters, all qualified and with several years of tea-tasting expertise, evaluated five tea indexes (shape, soup color, aroma, taste, and leaf base). The shape, soup color, aroma, taste, and leaf base of the tea were scored on a 100-point scale. The overall sensory score was calculated using the following formula: total score = shape × 25% + soup color × 10% + aroma × 25% + taste × 30% + leaf base × 10%.

### 2.4. Electronic Nose Analysis

Volatile substances in the tea samples were measured using the PEN3 electronic nose (Airsense Analytics GmbH, Schwerin, Germany), following the method described by Roy et al. [10]. Ten grams of black tea samples were placed in a 250 mL glass beaker, and 10 mL of boiling water was added. The beaker was sealed with plastic wrap and left to stand for 30 min to allow gas equilibrium in the container. Measurements were conducted for 90 s, with a pre-sampling time of 5 s and a gas flow rate of 0.4 L/min.

### 2.5. HS-SPME-GC-MS Analysis

Headspace solid-phase microextraction (HS-SPME) followed by gas chromatography–mass spectrometry (GC-MS) was employed to analyze the volatile compounds in tea samples [11]. For analysis, 2 g of the powdered sample was placed in a 20 mL headspace vial with 5 µL of 50 µg/mL ethyl decanoate (≥99%, Sigma-Aldrich, Shanghai, China). The vial was sealed and heated to 50 °C for 30 min to allow the aroma components to volatilize. A solid-phase microextraction fiber (50/30 µm DVB/CAR/PDMS, Supelco, Bellefonte, PA, USA) was inserted to adsorb volatile compounds for 30 min, after which the fiber was immediately injected into the GC-MS port and held for 5 min. The SPME fiber was aged as per the manufacturer’s instructions prior to use.

The analysis was conducted using an Agilent HP-5MS column (30 m × 0.25 mm, 0.25 µm). The column temperature was programmed as follows: an initial temperature of 50 °C, held for 2 min; followed by heating to 80 °C at 2 °C/min, held for 4 min; then heated to 180 °C at 5 °C/min, held for 5 min; and finally heated to 220 °C at 10 °C/min, held for 10 min. High-purity helium was used as the carrier gas at a flow rate of 1.0 mL/min with splitless injection. The electron ionization was operated in electron impact mode, scanning ions in the range of 40–600 m/z. The ion source, quadrupole, and transmission line temperatures were set at 230 °C, 150 °C, and 280 °C, respectively.

### 2.6. Statistical Analysis

Volatile compounds were identified by referencing the NIST11 mass spectrometry database [12], retaining compounds with a similarity score greater than 70%. The compounds were further confirmed by consulting the relevant literature. Quantitative analysis was conducted using the internal standard method, with the content of each component calculated as the ratio of its peak area to that of the internal standard. Data were expressed as mean ± standard error. One-way analysis of variance (ANOVA) was conducted using SPSS 13, while heat maps, radar maps, principal component analysis (PCA), Venn diagrams, and orthogonal partial least squares discriminant analysis (OPLS-DA) were created using R3.3 software (Boston, MA, USA).

## 3. Results and Discussion

### 3.1. Sensory Evaluation and Electronic Nose Analysis of Lapsang Souchong Black Tea of Different Grades

**Figure 1:**
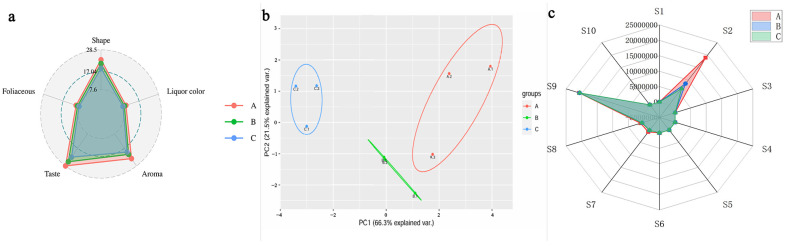
Sensory evaluation and electronic nose analysis of three grades of Lapsang Souchong black tea. (**a**) Radar chart displaying sensory scores. (**b**,**c**) Electronic nose evaluation results: (**b**) principal component analysis (PCA) illustrating the differentiation among grades; (**c**) radar chart depicting sensor responses. Grades A, B, and C correspond to special grade, first grade, and second grade, respectively.

The sensory evaluation results (Figure 1a) demonstrate a distinct quality ranking among the Lapsang Souchong black tea samples, with super-grade (A) tea receiving the highest ratings across all evaluated sensory characteristics, followed by first-grade (B) and second-grade (C). The assessed attributes—shape, soup color, aroma, taste, and leaf base—show a consistent ranking of A > B > C. The overall sensory scores corroborate this pattern: super-grade scored 94.30, first-grade 85.05, and second-grade 76.26. These findings indicate that higher-grade Lapsang Souchong black teas possess superior sensory qualities, including a more intense aroma, a well-balanced taste, and enhanced visual appeal in soup color and leaf integrity. The pronounced differences in sensory attributes underscore the critical role of quality control and grading in promoting consumer satisfaction with higher-grade teas.

Utilizing electronic nose technology to assess the volatile compounds present in the tea samples provided quantitative support for the sensory evaluation results. Electronic noses are commonly employed in food quality assessments and have shown efficacy in differentiating volatile profiles across various food products, including tea, wine, and coffee [3,13,14,15]. In this study, electronic nose analysis (Figure 1b,c) was utilized to compare the aroma profiles of the three grades of tea. Principal component analysis (PCA) revealed that the first principal component (PC1) accounted for 66.3% of the total variance, while the second principal component (PC2) explained an additional 21.5%. This analysis confirmed that volatile compounds, especially those related to aroma, play a pivotal role in distinguishing between tea grades. The clustering of data points along the first two principal components illustrates the strong relationship between aroma profiles and tea grades, reinforcing the idea that aroma significantly influences grade classification.

The radar chart (Figure 1c) further depicts the differences in sensor response values, which correlate with the concentrations of specific volatile compounds identified in the tea samples. Super-grade tea (A) displayed the highest sensor response values for components related to nitrogen oxides and terpenoids, followed by first-grade (B) and second-grade (C). This suggests that the higher levels of terpenoids and nitrogen-containing compounds in the super-grade tea contribute to its superior aroma profile, aligning with previous research identifying terpenoid content as a key factor in determining the quality of black tea [16,17]. Terpenoids, recognized for their floral, fruity, and herbal notes, significantly enhance the aromatic complexity of premium black teas. The diminished sensor responses in lower-grade teas imply that these important volatile compounds are present in lesser quantities, resulting in a less complex and less appealing aroma.

In summary, the sensory evaluation combined with electronic nose analysis offers a comprehensive view of the quality distinctions among the different grades of Lapsang Souchong black tea. The observed correlation between sensory attributes and the concentrations of volatile compounds emphasizes the value of both subjective (sensory) and objective (electronic nose) assessments in tea quality grading. The electronic nose’s ability to accurately identify key aroma compounds, such as terpenoids, establishes it as a valuable tool in the tea industry for quality control and grade differentiation.

### 3.2. Volatiles in Lapsang Souchong Black Tea of Different Grades

**Figure 2:**
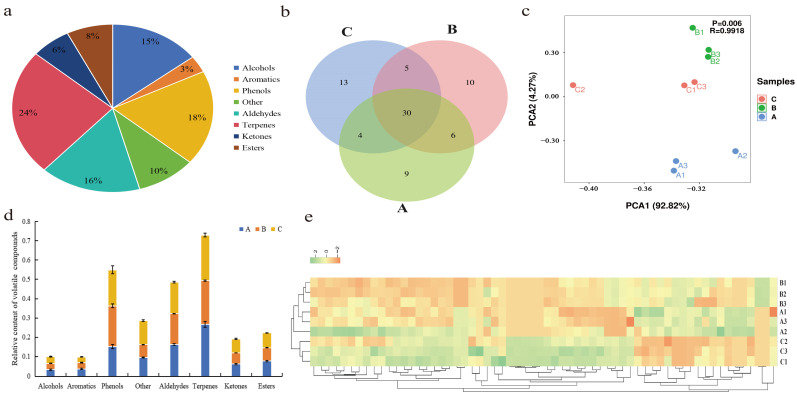
Analysis of volatile compounds and their classification in three grades of Lapsang Souchong black tea: (**a**) Doughnut chart illustrating the distribution of volatile compounds across all samples. (**b**) Venn diagram comparing the presence of compounds in grades A, B, and C. (**c**) Principal component analysis (PCA) showcasing the variance among the grades. (**d**) Relative abundance of various volatile compounds in grades A, B, and C. (**e**) Heatmap representation of the normalized relative content of each volatile compound, with rows corresponding to tea samples and columns to individual volatile components. Grades A, B, and C denote special grade, first grade, and second grade, respectively.

A total of 73 volatile compounds were identified in the various grades of Lapsang Souchong black tea (Tables S1–S3, Figures S1–S3). These compounds encompassed alcohols (n. 7), arenes (n. 4), phenols (n. 13), aldehydes (n. 7), terpenes (n. 12), ketones (n. 5), esters (n. 3), and others (n. 22) (Figure 2a). In terms of the variety of aromatic components, the order was C > B > A (Figure 2b). PCA analysis indicated differences in the aroma components of the Lapsang Souchong black tea samples across grades (Figure 2c). Principal component 1 accounted for 92.82% of the variation, while principal component 2 explained 4.27%, with R = 0.9918 and *p* = 0.006, reaching significance. Among the volatile components detected, terpenoids had the highest total relative content, followed by phenols and aldehydes (Figure 2d). Heat map analysis (Figure 2e) revealed that terpenes, alcohols, and phenols were significantly enriched in super-grade tea (A). Terpenes typically contribute floral, fruity, and woody notes [18]. Key compounds like Geraniol and D-Limonene possess floral or floral–fruity aromas [18,19]. Alcohols often present green, woody, and floral scents [20,21], with compounds such as 2-Furanmethanol providing strong woody–floral aromas [22].

In contrast, first-grade tea (B) showed a notable presence of terpenes such as α-Terpineol and Citral, all associated with floral and fruity aromas. Second-grade tea (C) exhibited a significant quantity of phenolic compounds, including various phenols known for their antioxidant and anti-inflammatory properties, which can enhance the complexity of tea aromas [23]. These phenolic compounds are primarily generated during the oxidation of polyphenols during fermentation, contributing to the flavor and aroma profiles of black tea [24].

Overall, the highest concentration of terpenoid compounds was found in Lapsang Souchong black tea, followed by phenols and aldehydes, which are generally linked to fermentation and floral characteristics. The analysis highlighted significant differences in the aroma components of Lapsang Souchong black tea across grades, particularly in the presence of floral and fruity terpenoid compounds. Further investigation is warranted to identify key differential volatile compounds in Lapsang Souchong black tea of varying grades.

### 3.3. Pairwise Comparison of Volatiles in Lapsang Souchong Black Tea of Different Grades

**Figure 3:**
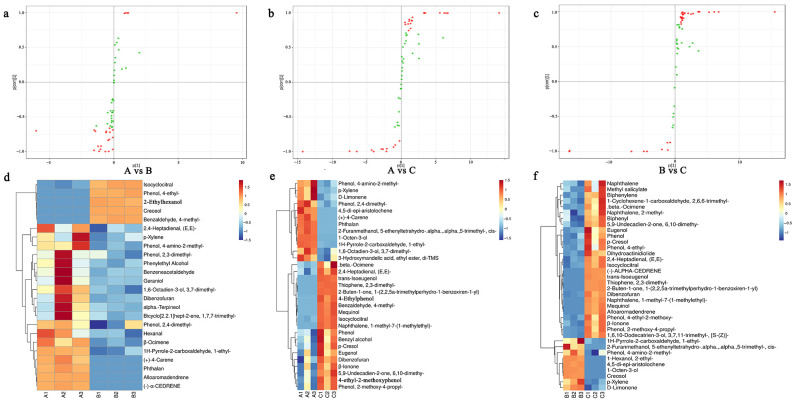
Pairwise analysis of volatile metabolites in three grades of Lapsang Souchong black tea: (**a**–**c**) OPLS-DA analysis illustrating the differences between (**a**) grades A and B, (**b**) grades A and C, and (**c**) grades B and C; red dots indicate metabolites with significant differences, while green dots represent those without significant differences. (**d**–**f**) Heatmaps showing the volatile compound profiles for (**d**) A compared to B, (**e**) A compared to C, and (**f**) B compared to C. Differential metabolites were identified based on their variable importance in projection (VIP > 1).

The pairwise comparison of Lapsang Souchong black tea across different grades revealed 52 differential compounds (Table S4), including six alcohols, three arenes, four aldehydes, 11 phenols, 12 terpenes, three ketones, two esters, and 11 others. There were a total of 24 differential substances in A vs. B (20 up-regulated and 4 down-regulated), including seven terpenes, five phenols, two alcohols, four aldehydes, one aromatic hydrocarbon, and five others (Figure 3a). There are a total of 31 differential substances in A vs. C (19 up-regulated and 12 down-regulated), including three alcohols, one aromatic hydrocarbon, nine phenols, four terpenes, two aldehydes, three ketones, and eight others (Figure 3b). There were a total of 36 differential substances in B vs. C (9 up-regulated and 27 down-regulated), including four alcohols, three aromatic hydrocarbons, nine phenols, seven terpenes, one aldehyde, three ketones, one ester, and eight others (Figure 3c). There were fewer differential substances between A vs. B. Among them, 2-ethylhexanol, creosol, benzaldehyde-4-methyl- and phenol-4-ethyl- are less in A than in B (Figure 3d). The typical characteristics of these substances were sweet, woody, fruity, and slightly floral [25]. However, A contains more terpene and aldehyde substances with fruity and floral aromas, so the aroma score in the sensory evaluation score of A is higher. In addition, Figure 3e,f show that C contains a large number of phenolic substances such as *p*-cresol, 2-Methoxy-4-ethylphenol, 4-ethyl-2-methoxyphenol, eugenol, and trans-Isoeugenol with floral, spicy, and smoky aromas. Li et al. [7] research shows that smoking was conducive to the accumulation of phenolic substances in Lapsang Souchong black tea. This may be the reason why the aroma score of C was not as high as that of A and B.

In conclusion, A contains a large number of terpene and aldehyde substances with fruity and floral aromas, while C also contains a large number of phenolic substances with floral and spicy aromas. Therefore, in the sensory evaluation, A has the highest score, and C has the lowest score.

### 3.4. Screening of Key Differential Volatiles

**Figure 4:**
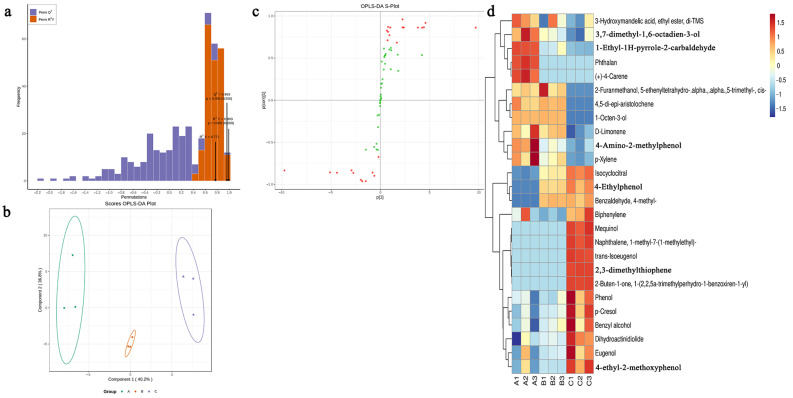
Identification of differentially volatile metabolites among grades A, B, and C: (**a**) OPLS-DA models assessing the goodness of fit for grades A, B, and C. (**b**) Validation of the OPLS-DA analysis for the three grades of Lapsang Souchong black tea. (**c**) Loading plot from the OPLS-DA analysis highlighting the volatile metabolites, where red dots indicate significant differences and green dots indicate non-significant differences. (**d**) Heatmap representing the differential metabolites across the three grades.

The results of analyzing the differential volatiles in Lapsang Souchong black tea by OPLS-DA show that during the construction of the OPLS-DA model, after 200 random simulations, the goodness-of-fit (R^2^Y = 0.993, *p* < 0.005) and predictability (Q^2^ = 0.963, *p* < 0.005) of the model both reach significant levels (Figure 4a). It can be seen that the OPLS-DA model can effectively distinguish different-grade Lapsang Souchong black tea samples, and the model construction meets the requirements and can be used for further analysis. The analysis results of the scores plot of the OPLS-DA models of A, B, and C show (Figure 4b) that the three grades can be effectively distinguished in different regions. The first principal component explains 40.2% of the differences, and the second principal component explains 36.8% of the differences. This indicates that there are differences in the aromas of Lapsang Souchong black tea of the three grades. Further, the S-Plot of the constructed OPLS-DA model was analyzed to obtain the variable importance in projection values (VIP values) of different volatiles and obtain the key differential volatiles. The analysis results show (Figure 4c) that a total of 26 key differential volatiles were obtained (Table S5). According to the classification of the obtained key differential volatiles, there were three alcohols, eight phenols, four terpenes, and one each of ketones, esters, aldehydes, and arenes. A contains abundant 3-Hydroxymandelic acid, ethyl ester, 3,7-dimethyl-1,6-octadien-3-ol, 1-Ethyl-1H-pyrrole-2-carbaldehyde, Phthalan, (+)- 4-Carene, D-Limonene with sweet, floral, and fruity aromas [26,27,28,29,30]. B contains abundant 2-Furanmethanol, 5-ethenyltetrahydro-α,α,5-trimethyl-, cis-,4,5-di-epi-aristolochene, 1-Octen-3-ol with earthy, floral, sweet, woody, and green aromas [21,25]. However, its aroma characteristics are not as elegant as those in A, so the aroma evaluation score of A is higher than that of B. C contains 3,7-dimethyl-1,6-octadien-3-ol, 2,3-dimethylthiophene, Dihydroactinidiolide, Naphthalene-1-methyl-7-(1-methylethyl)- with green, herbal scent, fruity, and slightly sweet aromas [31,32,33], and at the same time, C contains 4-ethyl-2-methoxyphenol with a rich smoky flavor during the smoking and baking process of Lapsang Souchong black tea.

There were significant differences in the key differential volatiles of the national standard samples of Lapsang Souchong black tea of the three grades. The main sweet, floral, and fruity aromas in A are mainly attributed to compounds such as D-Limonene, 3,7-dimethyl-1,6-octadien-3-ol, and 3-Hydroxymandelic acid,ethyl ester. 2-Furanmethanol, 5-ethenyltetrahydro-α,α,5-trimethyl-, cis-,4,5-di-epi-aristolochene, 1-Octen-3-ol mainly contribute to the floral, sweet, and woody aromas in B. The large amount of smoky substances, such as 4-ethyl-2-methoxyphenol in C, may lead to a decrease in its floral and fruity aromas.

## 4. Conclusions

The aroma characteristics of Lapsang Souchong black tea of different grades in this study were completely in line with the descriptions in Chinese national standards. The results of the sensory evaluation show that as the grade of Lapsang Souchong black tea decreases, the scores of all indicators decline. The results of electronic nose analysis show that the terpenoid substances in A are higher than those in B and C. There were significant differences in the key differential volatiles of Lapsang Souchong black tea of the three grades. Compounds such as D-Limonene, 3,7-dimethyl-1,6-octadien-3-ol, and 3-Hydroxymandelic acid, ethyl ester show a relatively high abundance in A. And 2-Furanmethanol-5-ethenyltetrahydro-α,α,5-trimethyl-cis-, 4,5-di-epi-aristolochene, 1-Octen-3-ol are related to the floral, sweet, woody, green characteristics of B. 3,7-dimethyl-1,6-octadien-3-ol, 2,3-dimethylthiophene, dihydroactinidiolide, Naphthalene-1-methyl-7-(1-methylethyl)- in C are related to its green, herbal scent, and fruity aroma. Meanwhile, C contains a relatively high amount of smoky-flavored substance 4-ethyl-2-methoxyphenol, which was an important factor affecting its aroma score. These differential metabolites were unique markers for characterizing and distinguishing Lapsang Souchong black tea of different grades. This finding has greatly enhanced our understanding of the main volatile compounds inherent in Lapsang Souchong black tea of different grades and provides a scientific basis for understanding the grade classification of Chinese national standard samples.

## Data Availability

The original contributions presented in the study are included in the article/Supplementary Materials; further inquiries can be directed to the corresponding author.

## Supplementary Materials

The following supporting information can be downloaded at: https://www.mdpi.com/article/10.3390/foods13213433/s1. Table S1 Aroma components in Lapsang Souchong black tea of special grade. Table S2 Aroma components in Lapsang Souchong black tea of the first grade. Table S3 Aroma components in Lapsang Souchong black tea of the second grade. Table S4 Two-by-two comparison analysis of volatile compounds in Lapsang souchong black tea. Table S5 Key differential volatile mass spectra. Figure S1 Chromatogram of aroma components of special grade Lapsang Souchong black tea. Figure S2 Chromatogram of aroma components of first grade Lapsang Souchong black tea. Figure S3 Chromatogram of aroma components of second grade Lapsang Souchong black tea.
