# Supplementary material for: Combined Analysis of Grade Differences in Lapsang Souchong Black Tea Using Sensory Evaluation, Electronic Nose, and HS-SPME-GC-MS, Based on Chinese National Standards
Source: Foods. 2024 Oct 28;13(21):3433. doi: 10.3390/foods13213433 (PMC11545444; doi:10.3390/foods13213433)
Supplement: Supplementary file 1 [file foods-13-03433-s001.zip › foods-3262667-supplementary.pdf]

Table S1 Aroma components in Lapsang Souchong black tea of special grade

Compound number	Retention time (min)	Peak area (Ab*s)	Matched item name	CAS number
1	3.799	3952264	Hexanal	000066-25-1
2	5.04	1891117	2-Hexenal, (E)-	006728-26-3
3	5.11	4570638	2-Hexenal, (E)-	006728-26-3
4	5.492	4268987	p-Xylene	000106-42-3
5	8.8	13097495	Benzaldehyde	000100-52-7
6	9.996	2202943	Phenol	000108-95-2
7	10.123	4974997	5-Hepten-2-one, 6-methyl-	000110-93-0
8	11.256	2212767	2,4-Heptadienal, (E,E)-	004313-03-5
9	11.904	776099	p-Cymene	000099-87-6
10	12.114	1669029	D-Limonene	005989-27-5
11	12.496	4689200	Benzyl alcohol	000100-51-6
12	12.941	5976708	Benzeneacetaldehyde	000122-78-1
13	13.266	6040129	Phenol, 4-amino-2-methyl-	002835-96-3
14	13.819	3585736	Phenol, 2-methyl-	000095-48-7
15	14.258	1610500	Phthalan	000496-14-0
16	15.066	3919455	p-Cresol	000106-44-5
17	15.563	12156395	2-Furanmethanol, 5-ethenyltetrahydro-.alpha.,.alpha.,5-trimethyl-, cis-	005989-33-3
18	15.912	2234975	3,5-Octadien-2-one, (E,E)-	030086-02-3
19	16.326	13103855	1,6-Octadien-3-ol, 3,7-dimethyl-	000078-70-6
20	16.593	11000831	Hotrienol	020053-88-7
21	17.039	6795305	Phenylethyl Alcohol	000060-12-8
22	17.929	778157	3-Hydroxymandelic acid, ethyl ester, di-TMS	1000071-88-9
23	18.578	1418916	Benzyl nitrile	000140-29-4
24	19.265	1306595	Phenol, 2,4-dimethyl-	000105-67-9
25	19.329	2205893	Phenol, 2,4-dimethyl-	000105-67-9
26	20.162	875920	2H-Pyran-3-ol, 6-ethenyltetrahydro-2,2,6-trimethyl-	014049-11-7
27	20.34	2455240	Phenol, 2,3-dimethyl-	000526-75-0
28	20.557	4790764	Naphthalene	000091-20-3
29	21.136	1964080	α-Terpineol	000098-55-5
30	21.257	15607250	Methyl salicylate	000119-36-8
31	21.498	1776315	1,3-Cyclohexadiene-1-carboxaldehyde, 2,6,6-trimethyl-	000116-26-7
32	22.376	2509233	1-Cyclohexene-1-carboxaldehyde, 2,6,6-trimethyl-	000432-25-7
33	22.713	552785	Bicyclo[2.2.1]hept-2-ene,	000464-17-5

			1,7,7-trimethyl-	
34	22.796	1414899	(+)-4-Carene	029050-33-7
35	22.993	574940	n-Valeric acid cis-3-hexenyl ester	035852-46-1
36	23.878	35598819	Geraniol	000106-24-1
37	24.38	2742192	Citral	005392-40-5
38	24.628	5300023	Phenol, 4-ethyl-2-methoxy-	002785-89-9
39	24.966	711236	Naphthalene, 1-methyl-	000090-12-0
40	25.544	1447311	Naphthalene, 1-methyl-	000090-12-0
41	27.479	524452	Eugenol	000097-53-0
42	27.828	937804	Phenol, 2-methoxy-4-propyl-	002785-87-7
43	28.096	654625	Biphenyl	000092-52-4
44	28.369	1319188	Hexanoic acid, 3-hexenyl ester, (Z)-	031501-11-8
45	28.452	707501	2,6-Octadien-1-ol, 3,7-dimethyl-, (Z)-	000106-25-2
46	29.082	741363	4,5-di-epi-aristolochene	1000374-17-1
47	29.915	960109	α-Ionone	000127-41-3
48	30.38	1029351	Biphenylene	000259-79-0
49	30.761	1347768	5,9-Undecadien-2-one, 6,10-dimethyl-	000689-67-8
50	31.875	4060638	trans-β-Ionone	000079-77-6
51	33.446	568243	2(4H)-Benzofuranone, 5,6,7,7a-tetrahydro-4,4,7a-trimethyl-	015356-74-8
52	35.011	1016916	1,6,10-Dodecatrien-3-ol, 3,7,11-trimethyl-, (E)-	040716-66-3

Table S2 Aroma components in Lapsang Souchong black tea of the first grade

Compound number	Retention time (min)	Peak area (Ab*s)	Matched item name	CAS number
1	3.818	4165345	Hexanal	000066-25-1
2	5.053	2029116	2-Hexenal, (E)-	006728-26-3
3	5.116	3982005	2-Hexenal, (E)-	006728-26-3
4	5.498	2478146	o-Xylene	000095-47-6
5	8.806	13679671	Benzaldehyde	000100-52-7
6	9.773	811265	1-Octen-3-ol	003391-86-4
7	10.002	2543427	Phenol	000108-95-2
8	11.262	1479872	2,4-Heptadienal, (E,E)-	004313-03-5
9	12.108	1414835	D-Limonene	005989-27-5
10	12.293	1597306	1-Hexanol, 2-ethyl-	000104-76-7
11	12.496	5496296	Benzyl alcohol	000100-51-6
12	12.942	4843373	Benzeneacetaldehyde	000122-78-1
13	13.279	3206343	1H-Pyrrole-2-carboxaldehyde, 1-ethyl-	002167-14-8
14	13.82	3766027	Phenol, 2-methyl-	000095-48-7
15	14.265	1593688	Benzaldehyde, 3-methyl-	000620-23-5
16	14.615	8932737	2-Furanmethanol, 5-ethenyltetrahydro-.alpha.,.alpha.,5-tri	005989-33-3

			methyl-, cis-	
17	15.066	4594311	p-Cresol	000106-44-5
18	15.569	11519339	2-Furanmethanol, 5-ethenyltetrahydro-.alpha.,.alpha.,5-tri methyl-, cis-	005989-33-3
19	15.913	2200117	3,5-Octadien-2-one, (E,E)-	030086-02-3
20	16.326	11769187	1,6-Octadien-3-ol, 3,7-dimethyl-	000078-70-6
21	16.593	8255562	1,5,7-Octatrien-3-ol, 3,7-dimethyl-	029957-43-5
22	17.032	6423255	Phenylethyl Alcohol	000060-12-8
23	17.923	628111	3-Hydroxymandelic acid, ethyl ester, di-TMS	1000071-88-9
24	18.578	686254	Benzyl nitrile	000140-29-4
25	19.265	1507202	Phenol, 3,5-dimethyl-	000108-68-9
26	19.329	1341189	Phenol, 2,4-dimethyl-	000105-67-9
27	20.162	746256	2H-Pyran-3-ol, 6-ethenyltetrahydro-2,2,6-trimethyl-	014049-11-7
28	20.341	2464279	Phenol, 2,3-dimethyl-	000526-75-0
29	20.557	4985573	Naphthalene	000091-20-3
30	21.136	1585355	L-.alpha.-Terpineol	010482-56-1
31	21.257	15159035	Methyl salicylate	000119-36-8
32	21.492	1781690	1,3-Cyclohexadiene-1-carboxaldehyde, 2,6,6-trimethyl-	000116-26-7
33	21.88	663229	Decanal	000112-31-2
34	22.37	2473829	1-Cyclohexene-1-carboxaldehyde, 2,6,6-trimethyl-	000432-25-7
35	22.714	483498	Bicyclo[2.2.1]hept-2-ene, 1,7,7-trimethyl-	000464-17-5
36	22.79	1195759	.beta.-Myrcene	000123-35-3
37	22.904	852650	2-Propenoic acid, 6-methylheptyl ester	054774-91-3
38	23.261	2126045	2,4-Dimethoxytoluene	038064-90-3
39	23.871	29721711	Geraniol	000106-24-1
40	24.38	3124769	Citral	005392-40-5
41	24.966	1253350	Naphthalene, 2-methyl-	000091-57-6
42	25.545	1669513	Naphthalene, 2-methyl-	000091-57-6
43	27.472	702755	Eugenol	000097-53-0
44	27.619	632412	2-Cyclopenten-1-one, 3-methyl-2-(2,4-pentadienyl)-, (Z)-	022610-79-3
45	27.822	1171240	Phenol, 2-methoxy-4-propyl-	002785-87-7
46	28.096	953131	Biphenyl	000092-52-4
47	28.369	1529465	Hexanoic acid, 3-hexenyl ester, (Z)-	031501-11-8
48	28.452	845369	2,6-Octadien-1-ol, 3,7-dimethyl-, (Z)-	000106-25-2
49	29.082	1008043	4,5-di-epi-aristolochene	1000374-17-1
50	29.909	1107732	.alpha.-Ionone	000127-41-3
51	30.38	951043	Biphenylene	000259-79-0

52	30.761	1435006	5,9-Undecadien-2-one, 6,10-dimethyl-	000689-67-8
53	31.875	4326264	trans-.beta.-Ionone	000079-77-6
54	32.715	521154	Dibenzofuran	000132-64-9
55	33.44	659565	2(4H)-Benzofuranone, 5,6,7,7a-tetrahydro-4,4,7a-trimethyl-	015356-74-8
56	35.005	958131	1,6,10-Dodecatrien-3-ol, 3,7,11-trimethyl-, [S-(Z)]-	000142-50-7

Table S3 Aroma components in Lapsang Souchong black tea of the second grade

Compound number	Retention time (min)	Peak area (Ab*s)	Matched item name	CAS number
1	3.818	3492500	Hexanal	000066-25-1
2	5.053	6388318	2-Hexenal, (E)-	006728-26-3
3	5.504	1588550	p-Xylene	000106-42-3
4	8.806	11372336	Benzaldehyde	000100-52-7
5	9.996	3058173	Phenol	000108-95-2
6	11.256	2995576	2,4-Heptadienal, (E,E)-	004313-03-5
7	12.114	1083634	D-Limonene	005989-27-5
8	12.496	5909132	Benzyl alcohol	000100-51-6
9	12.941	4600901	Benzeneacetaldehyde	000122-78-1
10	13.279	2439730	Phenol, 4-amino-2-methyl-	002835-96-3
11	13.819	3933027	Phenol, 2-methyl-	000095-48-7
12	14.252	1504517	Benzaldehyde, 3-methyl-	000620-23-5
13	15.066	5386735	p-Cresol	000106-44-5
14	15.569	11242665	Mequinol	000150-76-5
15	15.919	2240232	3,5-Octadien-2-one, (E,E)-	030086-02-3
16	16.326	9963069	1,6-Octadien-3-ol, 3,7-dimethyl-	000078-70-6
17	16.593	7198632	1,5,7-Octatrien-3-ol, 3,7-dimethyl-	029957-43-5
18	17.039	6578847	Phenylethyl Alcohol	000060-12-8
19	17.929	640970	3-Hydroxymandelic acid, ethyl ester, di-TMS	1000071-88-9
20	18.584	730538	Benzyl nitrile	000140-29-4
21	19.265	1796566	Phenol, 3,5-dimethyl-	000108-68-9
22	19.335	2693417	Phenol, 2,5-dimethyl-	000095-87-4
23	20.162	812533	2H-Pyran-3-ol, 6-ethenyltetrahydro-2,2,6-trimethyl-	014049-11-7
24	20.245	980744	Phenol, 4-ethyl-	000123-07-9
25	20.347	2076401	Phenol, 2,3-dimethyl-	000526-75-0
26	20.557	5318438	Naphthalene	000091-20-3
27	21.142	1633723	L-.alpha.-Terpineol	010482-56-1
28	21.257	15765277	Methyl salicylate	000119-36-8
29	21.498	1305494	1,3-Cyclohexadiene-1-carboxaldehyde, 2,6,6-trimethyl-	000116-26-7
30	21.886	588335	Decanal	000112-31-2

31	22.37	2964217	1-Cyclohexene-1-carboxaldehyde, 2,6,6-trimethyl-	000432-25-7
32	22.72	520086	Bicyclo[2.2.1]hept-2-ene, 1,7,7-trimethyl-	000464-17-5
33	22.796	1511389	.beta.-Ocimene	013877-91-3
34	23.261	2270917	2,3-Dimethoxytoluene	004463-33-6
35	23.871	29800183	Geraniol	000106-24-1
36	24.38	2938880	Citral	005392-40-5
37	24.628	8075156	Phenol, 4-ethyl-2-methoxy-	002785-89-9
38	24.966	1326409	Naphthalene, 2-methyl-	000091-57-6
39	25.544	1975636	Naphthalene, 2-methyl-	000091-57-6
40	27.472	937220	Eugenol	000097-53-0
41	27.822	1652277	Phenol, 2-methoxy-4-propyl-	002785-87-7
42	28.102	1164415	Biphenyl	000092-52-4
43	28.369	1463261	Hexanoic acid, 3-hexenyl ester, (Z)-	031501-11-8
44	28.458	952187	2,6-Octadien-1-ol, 3,7-dimethyl-, (Z)-	000106-25-2
45	29.082	994471	Alloaromadendrene	025246-27-9
46	29.909	1578669	.alpha.-Ionone	000127-41-3
47	30.38	1441719	Biphenylene	000259-79-0
48	30.761	2321576	5,9-Undecadien-2-one, 6,10-dimethyl-, (E)-	003796-70-1
49	31.875	6192556	trans-.beta.-Ionone	000079-77-6
50	32.721	879819	Dibenzofuran	000132-64-9
51	33.44	1159786	2(4H)-Benzofuranone, 5,6,7,7a-tetrahydro-4,4,7a-trimethyl-	015356-74-8
52	35.005	1606264	1,6,10-Dodecatrien-3-ol, 3,7,11-trimethyl-, [S-(Z)]-	000142-50-7
53	35.539	590613	Naphthalene, 1-methyl-7-(1-methylethyl)-	000490-65-3

Table S4 Two-by-two comparison analysis of volatile compounds in Lapsang souchong black tea

Kinds	Numbers
Alcohols	6
Aromatics	3
Phenols	11
Other	11
Aldehydes	4
Terpenes	12
Ketones	3
Esters	2

Table S5 Key differential volatile mass spectra

Name	Mass spectra
p-Xylene	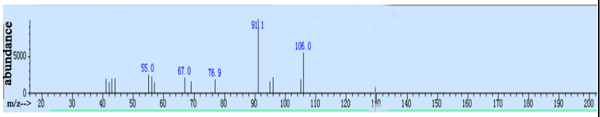
1-Octen-3-ol	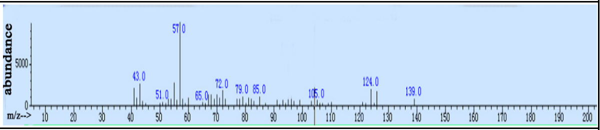
Phenol	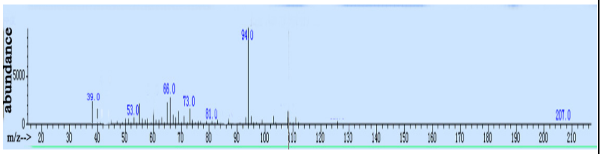
D-Limonene	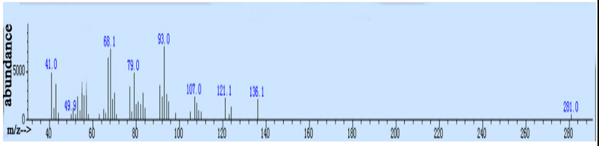
Benzl alcohol	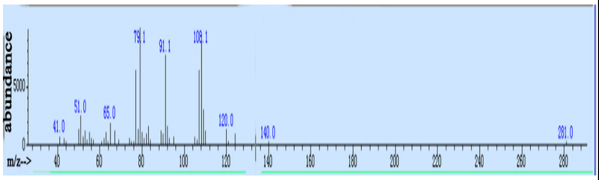
4-Amino-2-methylphenol	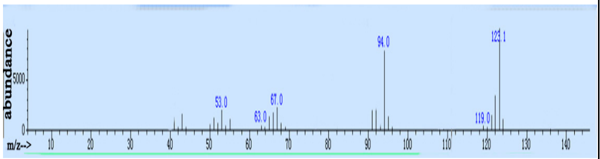
1-Ethyl-1H-pyrrole-2-carbaldehyde	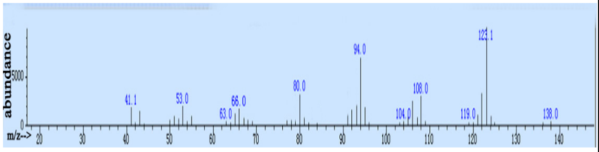
Benzaldehyde, 4-methyl-	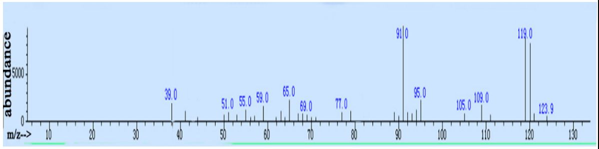
Phthalan	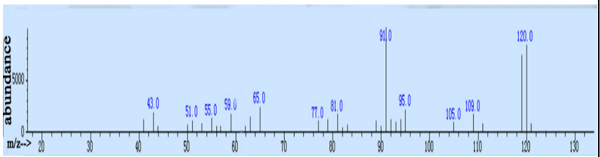
p-Cresol	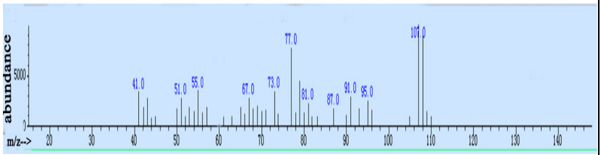

2-Furanmethanol, 5-ethenyltetrahydro-.α,α,5-trimethyl-, cis-	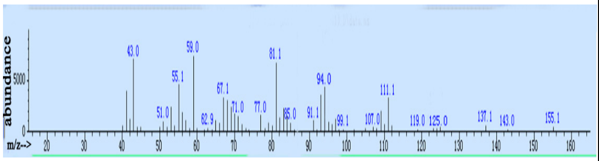
Mequinol	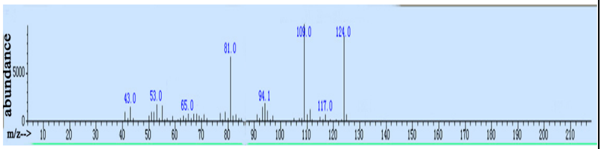
3,7-dimethyl-1,6-octadien-3-ol	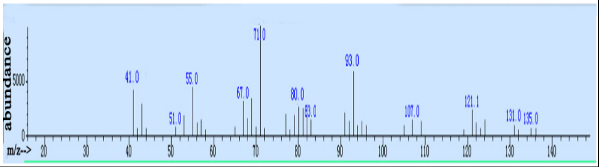
3-Hydroxymandelic acid, ethyl ester, di-TMS	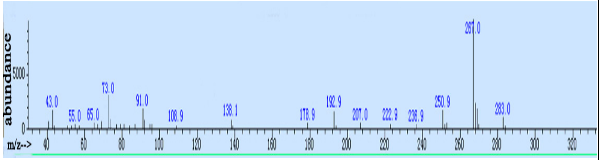
2,3-dimethylthiophene	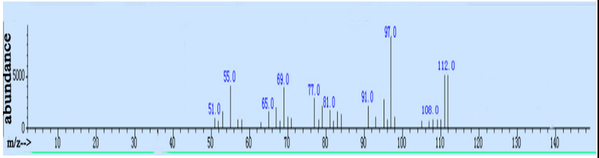
(+)-4-Carene	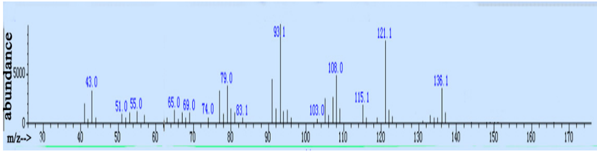
Isocyclocitral	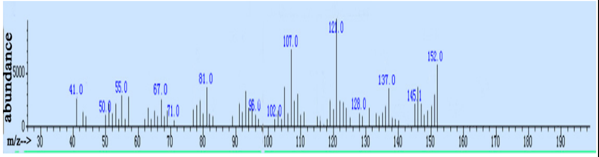
2-Methoxy-4-ethylphenol	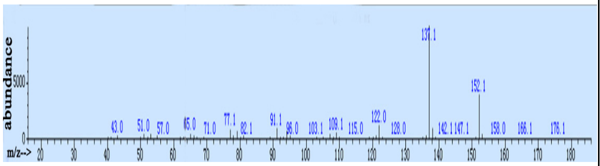
2-Buten-1-one, 1-(2,2,5a-trimethylperhydro-1-benzoxiren-1-yl)	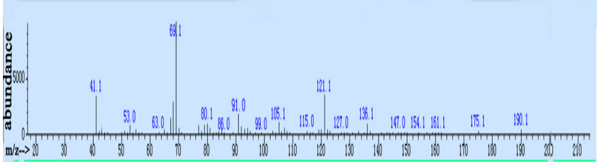
Eugenol	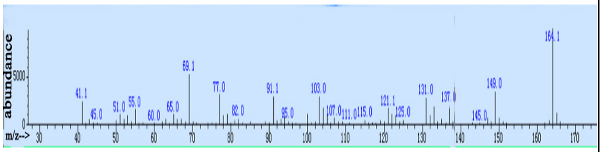
4-ethyl-2-methoxyphenol	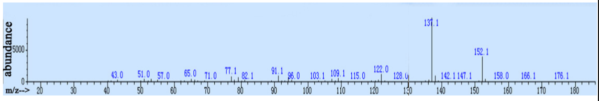

4,5-di-epi-aristolochene	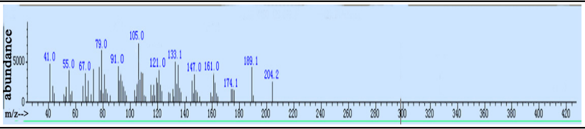
Biphenylene	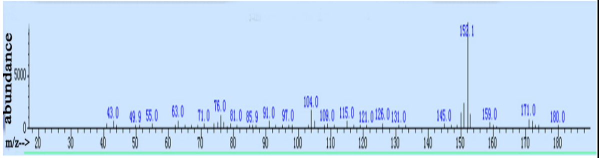
trans-Isoeugenol	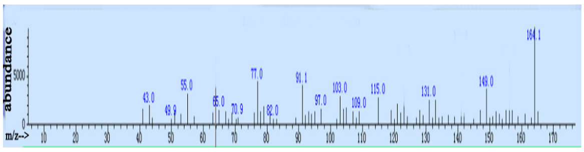
Dihydroactinidiolide	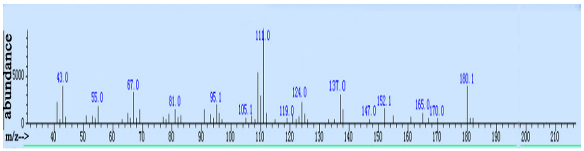
Naphthalene, 1-methyl-7-(1-methylethyl)-	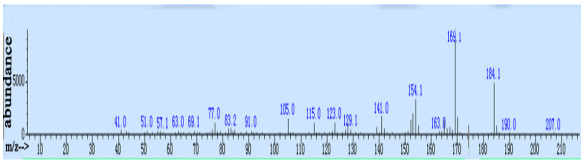

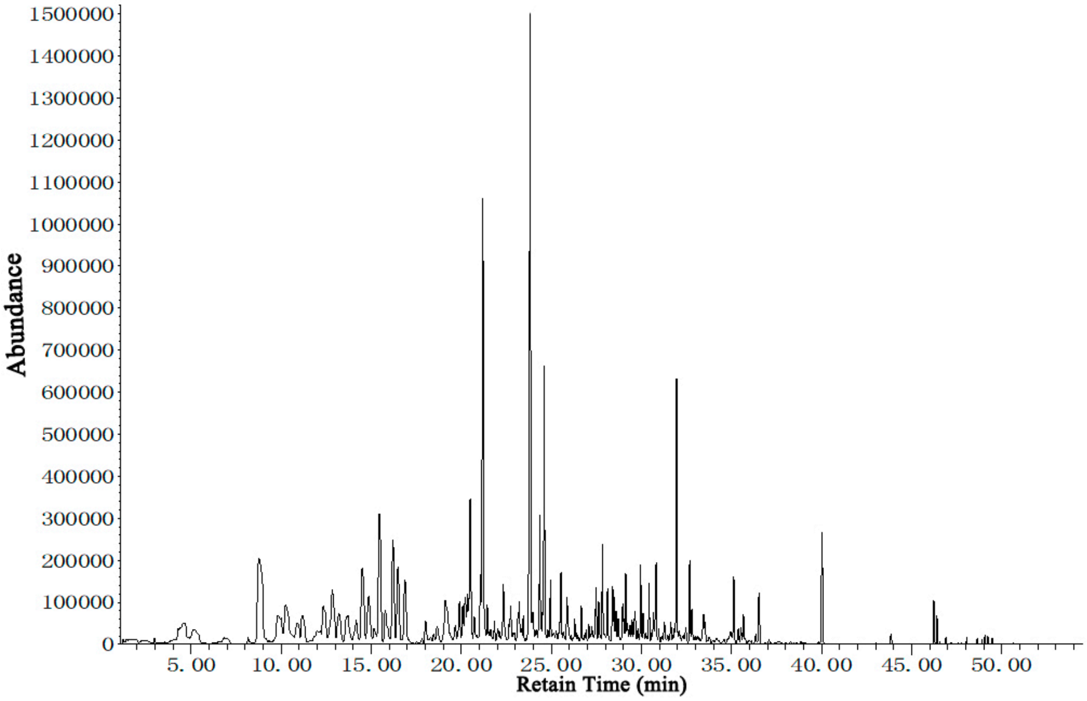

Figure S1 Chromatogram of aroma components of special grade Lapsang Souchong black tea

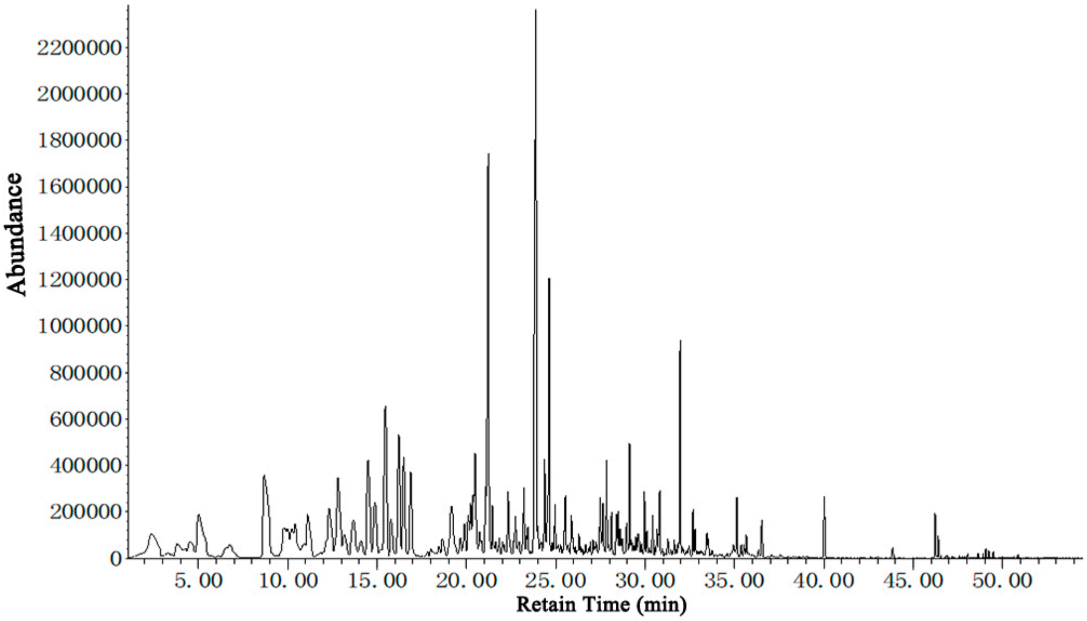

Figure S2 Chromatogram of aroma components of first grade Lapsang Souchong black tea

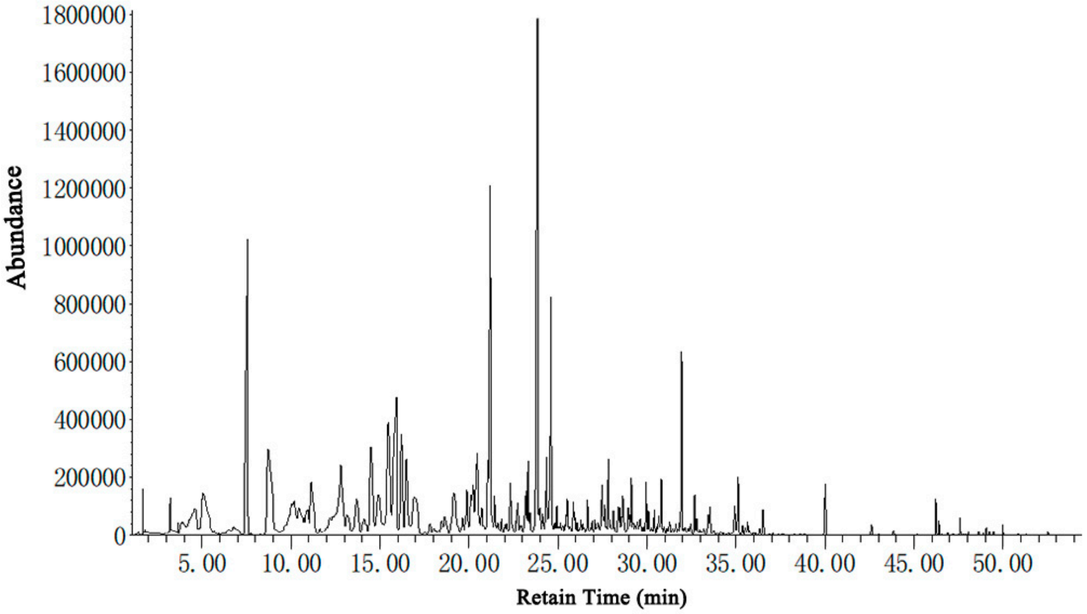

Figure S3 Chromatogram of aroma components of second grade Lapsang Souchong black tea
